# Glutathione Transferases: Potential Targets to Overcome Chemoresistance in Solid Tumors

**DOI:** 10.3390/ijms19123785

**Published:** 2018-11-28

**Authors:** Marija Pljesa-Ercegovac, Ana Savic-Radojevic, Marija Matic, Vesna Coric, Tatjana Djukic, Tanja Radic, Tatjana Simic

**Affiliations:** 1 Institute of Medical and Clinical Biochemistry, Faculty of Medicine, University of Belgrade, 11 000 Belgrade, Serbia; ana.savic-radojevic@med.bg.ac.rs (A.S.-R.); marija_opacic@yahoo.com (M.M.); coricmvesna@gmail.com (V.C.); tatjana.djukic@med.bg.ac.rs (T.D.); tatjana.simic@med.bg.ac.rs (T.S.); 2 Faculty of Medicine, University of Belgrade, 11 000 Belgrade, Serbia; tanjajevtic@gmail.com

**Keywords:** glutathione transferases, chemoresistance, protein-protein interaction, inhibitors, pro-drugs

## Abstract

Multifunctional enzymes glutathione transferases (GSTs) are involved in the development of chemoresistance, thus representing a promising target for a novel approach in cancer treatment. This superfamily of polymorphic enzymes exhibits extraordinary substrate promiscuity responsible for detoxification of numerous conventional chemotherapeutics, at the same time regulating signaling pathways involved in cell proliferation and apoptosis. In addition to upregulated GST expression, different cancer cell types have a unique GST signature, enabling targeted selectivity for isoenzyme specific inhibitors and pro-drugs. As a result of extensive research, certain GST inhibitors are already tested in clinical trials. Catalytic properties of GST isoenzymes are also exploited in bio-activation of specific pro-drugs, enabling their targeted accumulation in cancer cells with upregulated expression of the appropriate GST isoenzyme. Moreover, the latest approach to increase specificity in treatment of solid tumors is development of GST pro-drugs that are derivatives of conventional anti-cancer drugs. A future perspective is based on the design of new drugs, which would selectively target GST overexpressing cancers more prone to developing chemoresistance, while decreasing side effects in off-target cells.

## 1. Mechanisms of Chemoresistance

Chemoresistance is a multifactorial phenomenon and a common problem in cancer treatment. There are several mechanisms for cancer cells to acquire resistance to anti-cancer drugs [1,2]. The major ones include drug inactivation, induction of efflux transporters, inhibition of apoptosis, cell cycle and check points deregulation, acquired mutations and epigenetic alterations [2]. One of the most extensively studied mechanisms is anti-cancer drug inactivation by detoxification enzymes, especially glutathione transferases. In this line, a wide range of currently used conventional chemotherapeutics are recognized as substrates for glutathione transferases [1,3]. 

## 2. Glutathione Transferases in Cancer

Glutathione transferases (glutathione *S*-transferases or GSTs) are multifunctional enzymes involved in a number of catalytic and non-catalytic processes, still traditionally recognized as phase II cellular detoxification system enzymes. They are able to catalyze the nucleophilic addition of glutathione (GSH) to a wide variety of non-polar exogenous (chemical carcinogens, environmental pollutants and even antitumor agents) and endogenous compounds, yielding more water-soluble products, hence facilitating their elimination [4,5,6,7]. Due to the fact that reactions catalyzed by GSTs do not compulsorily result in detoxification of a foreign compound, sometimes GSTs are rather involved in xenobiotic bio-activation, resulting in even more reactive GSH-conjugate than the parent compound. This is especially true for certain mutagens, carcinogens and even some pro-drugs, which are metabolically activated in this way [8,9].

The overall functions of this set of cellular proteins (GSTome) may be classified into: (1) metabolism of xenobiotics and endogenous compounds, including intracellular binding and transport of hydrophobic compounds [10], catalysis of key steps in the synthesis of leukotrienes, prostaglandins [11] and steroid hormones [12], as well as the degradation of tyrosine [5], and inactivation and reduction of oxidative stress by-products [13] and (2) the regulation of cell signaling (such as protein-protein interactions with mitogen-activated protein kinases (MAPK)) [6,14,15,16]. 

According to their intracellular localization, GSTs are divided into three major families of proteins: cytosolic, mitochondrial and microsomal [5,17]. Based on their chemical, physical and structural properties, seven classes are recognized within cytosolic GSTs. Apart from observed variability between GST classes, a substantial genetic heterogeneity is found within classes, due to gene duplications, deletions and single nucleotide polymorphisms in both coding and non-coding gene regions [18]. Mentioned genetic variations have a direct impact on GST protein structure, function and expression, reshaping their substrate specificity and diversity as well, ultimately leading to complete lack or lowering of enzyme activity [14].

The vast majority of polymorphisms identified within genes encoding for cytosolic GSTs comprise single nucleotide polymorphisms (SNPs). Indeed, SNP leading to amino acid substitution from isoleucine (Ile) to valine (Val) [19] changes catalytic and regulatory properties of the GSTP1 enzyme [20], while *GSTA1* polymorphism is represented by three, apparently linked, SNPs: -567TOG, -69COT and -52GOA. These substitutions result in differential expression with lower transcriptional activation of the variant GSTA1*B (-567G, -69T, -52A) than common GSTA1*A allele (-567T, -69C, -52G) [21]. Amino acid substitution of Ala to Asp at position 140, as a result of SNP (C to A) in exon 4 of *GSTO1* gene (GSTO1*Ala140Asp), changes their deglutathionylase and thioltransferase activity [22,23,24]. Regarding *GSTO2* rs156697 polymorphism, SNP (A to G) leading to Asn to Asp substitution at position 142 (GSTO2*Asn142Asp) may be related to altered protein levels [25,26]. Functional significance of *GST* SNPs has recently been highlighted by Hollman et al. who suggested a classification of diseases highly related to SNPs found in GSTs, including cancers [18]. On the other hand, deletion polymorphisms of genes encoding for human cytosolic GSTM1 and GSTT1 are rather common in human populations. Approximately half of the population lacks GSTM1 enzyme activity, due to a homozygous deletion of the *GSTM1* gene [27] while in the case of *GSTT1*, gene homozygous deletion, with consequential lack of GSTT1 enzyme activity, is present in approximately 20% of Caucasians [28]. 

Although GSTs seem to be ubiquitously expressed, the expression of different GST genes may vary significantly between tissues, giving each organ a unique and complex GST profile [29]. This inter-individual variability in GST profile further affects the biotransformation capacity of certain tissue and the potential genotoxicity of certain carcinogens on that tissue. This variability is even more potentiated in cancer cells [29]. In general, GSTP1 over-expression seems to be a hallmark of proliferating cells in many solid tumors, including transitional cell carcinoma of urinary bladder [30,31], renal cell carcinoma [32,33], ovarian cancer [34,35], breast cancer [36,37] and colorectal cancer [38,39]. Regarding other GST classes, increased expression of GSTA1 is confirmed in colorectal cancer [39], GSTO1-1 is upregulated in transitional cell carcinoma [40], esophageal squamous cell carcinoma [41], pancreatic cancer [42], and breast cancer [43], while GSTM1 overexpression is observed in transitional cell carcinoma of urinary bladder [31], renal cell carcinoma [33] and breast cancer [44]. There is some evidence on differential expression of GSTM class (GSTM2-2 and GSTM4) and GSTP1 in osteosarcoma and soft tissue sarcoma patients [45,46,47]. Moreover, GSTs overexpression has also been identified in chemoresistant cancer cell lines, which has been attributed to induction of its expression during chemotherapy and a role in inhibiting apoptosis [48,49]. Since classical enzymatic functions of GSTs seem to coexist with their regulatory ones, giving them dual functionality, cytosolic GSTs are considered relevant when it comes to cancer development and progression, but also therapy resistance.

## 3. Catalytic Role of Glutathione Transferases in Detoxification and/or Bio-Activation of Anti-Cancer Drugs

**Figure 1:**
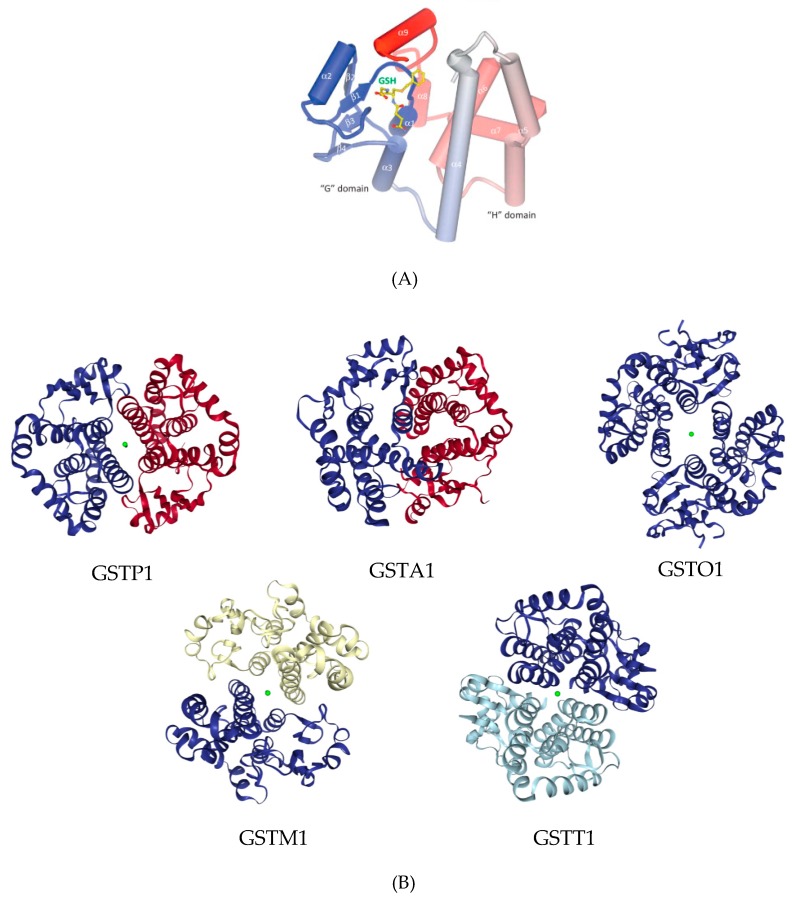
Glutathione transferases (GST) structure variability among classes: (**A**) Tertiary structure of GST enzyme, consisting of “G” domain for glutathione (GSH) binding, and “H” domain for hydrophobic substrates, adopted from Wu et al. [7], with the permission by Elsevier Ltd (Copyright 2012); (**B**) Crystal structures of human GSTs, adopted Protein Data Bank.

Despite the low sequence identity (<10%) amongst GST superfamily members, the tertiary and quaternary structures are remarkably consistent. All members of the GST superfamily contain an N-terminal thioredoxin-like fold and α-helical C-terminal region. Dimeric structure enhances protein stability and provides the active site with proper structure for catalysis. The position of the active site is well conserved in all catalytically active cytosolic GSTs, but still there are significant differences between classes (Figure 1) concerning the different reactions that are characteristically catalyzed. 

The active site is subdivided into the G site (within N-terminal domain) for GSH binding and H site (within C-terminal domain) which binds various hydrophobic and electrophylic substrates [50]. GST Alpha, Mu and Pi classes have accessible and open G-site, while G-site in the Theta and Zeta class is rather hidden and is not easily accessible to GSH [14]. Diversity of GSTs substrate specificities is due to different amino acids residues in the H-site of GST isoenzymes. GST Alpha, Mu, Pi and Sigma classes possess tyrosine in the active site [50] while GST Theta and Zeta classes possess serine residue [51,52] and GST Omega class a cysteine residue as a functional group [53]. 

Being known as enzymes of phase II of xenobiotic metabolism, their main and classic catalytic role is to conjugate a range of hydrophobic and electrophilic compounds, including many anti-cancer drugs and carcinogens, with GSH. These water-soluble GSH-conjugates [5] are further exported from the cell by membrane bound multi-drug resistant protein (MRP) efflux pumps [54] and excreted by bile or urine. In this way, secondary products of metabolism are detoxified; however, in some cases it results in formation of product even more toxic then xenobiotic itself. Moreover, GSTs possess antioxidant activity towards endogenously produced free radicals [14]. Even more, certain GSTs are able to conjugate the products of lipid peroxidation, such as 4-hydroxynonenal [5]. Interestingly, theta GST class is known to have a unique sulfatase activity [55]. Besides, some members of GSTs exhibit several other catalytic functions, such as thiol transferase activity, thiolysis and isomerization. Namely, GSTA, GSTM, GSTZ and GSTS exhibit isomerization catalytic activity [56,57]. Moreover, GSTA1-1, GSTA2-2, GSTM1-1 and GSTP1-1 are capable for metabolizing prostaglandins, PGA2 and PGJ2, which are recognized as inhibitors of cellular proliferation [58]. There is increasing evidence that GSTP class is also involved in glutathionylation, reversible formation of disulphide bonds between protein cysteinyl thiol and glutathione [59].

In comparison to other GSTs, omega class (GSTO) has its own range of enzymatic activities, including thioltransferase, dehydroascorbate reductase (DHAR) and monomethylarsenate reductase activities [60]. GSTO1-1 has been found to play a previously unappreciated role in the glutathionylation cycle that is emerging as significant mechanism regulating protein function. Namely, GSTO1-1 deglutathionylates proteins by forming mixed disulfides with GSH. Specific deglutathionylation by GSTO1-1 leads to the potential on/off regulation of protein function, while the polarity of the on/off switch is likely to be protein-specific. The capacity of GSTO1-1 to specifically deglutathionylate proteins [24] appears to be its primary physiological function and suggests a mechanism by which GSTO1-1 could potentially regulate cellular metabolism and signaling pathways that influence the growth and survival of cancer cells.

Some of the conventional anti-cancer drugs, such as chlorambucil [29,61,62,63], cyclophosphamide [29,63], melphalan [29,62,63,64], carmustine [29,55,62], cisplatin [65], busulfan [66], and thiotepa [29,63,67], are also substrates for GSTs and can be directly inactivated through conjugation reaction with glutathione (Table 1). It seems that alkylating agents are overrepresented among anti-cancer drugs which are GST substrates, due to the fact that they undergo well established GST-dependent drug conjugation reactions [68]. However, there are several possible ways in which GSTs might be responsible for chemoresistance towards anti-cancer drugs which are not known substrates for GSTs [1]. 

Being predominantly overexpressed GST isoenzyme in cancer cells, GSTP1 plays a significant role in resistance to chemotherapy as confirmed in pre-clinical data from cancer cell lines, but also in cancer patients [62,71,72]. Namely, its upregulated expression has been related to worse chemotherapeutic response to anti-cancer drugs such as cisplatin [73] and chlorambucil [74], recognized as GSTP1 substrates. On the other hand, inhibition of GSTP expression, through antisense cDNA, increases the cancer cell sensitivity to adriamicin, cisplatin, melphalan and etoposide due to decreased detoxification of mentioned drugs [75]. Besides GSTP1, overexpression of GSTA class has also been associated with the resistance to various alkylating agents [76] and doxorubicin [77,78]. In this line, GSTA1-1 overexpression seems to weaken the doxorubicin dependent depletion of glutathione, particularly in the H69 small cell lung cancer cell line, decreasing the extent of lipid peroxidation [78]. GSTM1 isoenzyme also detoxifies certain anti-cancer drugs, mostly including alkylating agents [62]. However, some other mechanism apart from GSH-conjugation might contribute to chemoresistance development. Namely, it has been shown that GSTP may influence doxorubicin resistance in tumor cells by the suppression of doxorubicin conversion to semiquinone free radical and subsequent production of superoxide anion radicals and peroxides [72,79]. Similarly, chemoresistance to anthracyclines was observed in cancer cells due to reduction of cellular ROS accomplished by antioxidant activity of both GSTP and GSTA [24,63]. 

## 4. Glutathione Transferases in Regulation of Signaling Pathways Involved in Cell Proliferation and Cell Death

**Figure 2:**
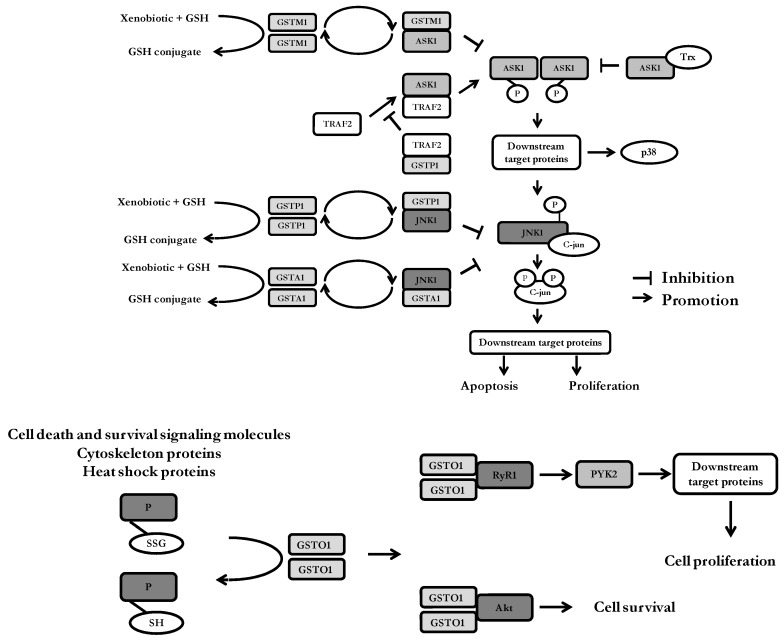
Dual functionality of GSTs in cancer: coexistence of catalytic and regulatory roles.

In addition to their catalytic role, there is some evidence which clearly indicate the involvement of several GSTs in the regulation of signaling pathways, by means of interactions with members of the mitogen-activated protein kinase (MAPK) signaling pathway (JNK-c- Jun N-terminal kinase, ASK- apoptosis signal-regulating kinase, Akt-protein kinase B) and certain receptors [6,15,16,24,80] (Figure 2).

The GSTP1:JNK1 protein-protein interaction was the first example of GST-mediated MAPK regulation discovered by Adler et al [81]. By forming this interaction, GSTP1 sequesters the JNK in a complex, inhibiting its activity and affecting the regulatory pathways that control cell proliferation and death [24,82]. Under physiological conditions, basal activity of JNK is essentially maintained at a low level. However, in response to different stimuli, the GSTP1:JNK1 complex dissociates, which in turn leads to the association of GSTP1 into oligomers. Now activated, JNK1 induces a chain of events, starting from the phosphorylation of c-Jun and results in the induction of AP-1-dependent target genes involved in cell proliferation, DNA repair and cell death [14,81]. Nevertheless, many studies implicate that the extent of JNK-activation inversely correlates with the expression level of GSTP1 [6]. In the case of an adaptor signaling protein, tumor necrosis factor receptor-associated factor 2 (TRAF2), a similar interaction with GSTP1 was observed. Specifically, TRAF2 mediates the signal transduction of different receptors and is required for the activation of the apoptosis signal- regulating kinase (ASK1) [83,84], which in turn activates both JNK and p38 signaling pathways. In that way, GSTP1 interaction with different signaling molecules is regulating the MAPK/JNK signaling cascade at multiple levels. It is noteworthy to mention that the catalytic activity of GSTP1 is not affected by the involvement in protein-protein interactions, suggesting that the active site of GTSP1 is not engaged in this process [6]. Overall, these findings can explain why overexpression of GSTP1-1 has extensively been linked with the resistance to apoptosis and chemoresistant phenotype of different solid cancers, even when certain anti-cancer drugs are not GSTP1 substrates [85]. Moreover, comparative protein-protein interaction studies revealed that in the case of common *GSTP1* polymorphism, haplotype GSTP1*C (*Val105/Val114*) is a better JNK inhibitor, hence with the greater anti-apoptotic effect than the haplotype GSTP1*A (*Ile105/Ala114*) [86].

- In addition to their catalytic role in detoxification of xenobiotics, GSTs are also involved in the regulation of cellular proliferation and apoptosis by the means of protein-protein interactions with signaling molecules. Regarding GSTM1, the same region of ASK1 seems to be engaged in protein-protein interactions with either GSTM1 or thioredoxin (Trx), suggesting the presence of both GSTM1:ASK1 and ASK1:Trx complexes under unstressed conditions. GSTP1 acts as negative regulator of JNK1, as well as TRAF2. Moreover, GSTP1:TRAF2 interaction prevents ASK1:TRAF2 interaction and, consequently, ASK1 activation. The structural homology between GSTA1 and GSTP1 may explain why GSTA1 can also suppress JNK1 signaling by a similar mechanism. Various types of cell stress can result in the disassociation of GSTs from the signaling molecules. Importantly, redox-sensitive dynamic equilibrium comprises catalytic homodimeric forms of GSTs, as well as its monomeric regulatory forms. ASK1—apoptosis signal—regulating kinase; JNK1-c-Jun N-terminal kinases; TRAF2—tumor necrosis factor receptor-associated factor 2; Trx—thioredoxin;
- GSTO1-1 deglutathionylates some cell death and survival signaling molecules, cytoskeleton and heat shock proteins by forming mixed disulfides with GSH. Specific deglutathionylation by GSTO1-1 leads to the potential protein-specific regulation of protein function. GSTO1-1 also interacts with the ryanodine receptor, RyR1 and promotes calcium release from the endoplasmic reticulum. Increased cytosolic calcium levels activate PYK2 leading to cell proliferation. GSTO1-1 interaction with Akt influences cell survival signaling pathways. RyR1—ryanodine receptor type 1; PYK2—proline-rich tyrosine kinase 2; Akt—protein kinase B.

GSTA1 also possesses the capacity of forming protein-protein complexes with JNK1, but it showed weaker JNK inhibitory activity. Namely, the homology between GSTA and GSTP family members may explain why GSTA1 can also suppress JNK1 signaling by a similar mechanism, caused by inflammatory cytokines or oxidative stress. Furthermore, it seems that enhanced GSTA1-1 expression significantly decreases the number of cells subjected to apoptosis due to inhibition of JNK1-dependent phosphorylation of c-jun and the activation of caspase 3 [87].

Complex between MAPK member, ASK1 and GSTM1, is found to be important for the maintenance of the normal level of p38 phosphorylation [88]. Namely, ASK1 belongs to upstream activator of JNK1 and p38 pathways, leading to cytokine and stress-induced apoptosis [89]. Environmental stress causes the disruption of GSTM1:ASK1 protein-protein interaction, leading to ASK1 activation, while GSTM1 accumulates into oligomers [90]. This dissociation results in a subsequent activation of JNK1 and p38-dependent signaling pathways, ultimately leading to stress-induced apoptosis. Indeed, in tumor tissue of clear cell renal cell carcinoma, ASK1 was co-immunoprecipitated with GSTM1 [91]. Similarly to GSTP1, this role of GSTM1 is shown to be independent of the GST enzyme activity [88]. 

It seems that several GSTs, such as GSTO1-1, GSTA1-1 and GSTM2-2 can modulate activity of ryanodine receptors (RyRs) (Figure 1B). The role of RyRs, a class of ligand-gated Ca^2+^ channels, is to release Ca^2+^ from intracellular stores in response to a range of intracellular and external stimuli [14]. Recently, signaling events involving interaction of GSTO1 with type 1 ryanodine receptor, RyR1 has been implicated in a signaling pathway that stimulates cancer stem cell enrichment during chemotherapy. Lu et al. reported increased GSTO1 expression in a HIF-dependent manner after exposure of breast cancer cells to chemotherapy. Consequently, GSTO1 activates RYR1, leading to activation of PYK2/SRC/STAT3 signaling [43]. In transitional cell carcinoma, GSTO1 co-immunoprecipitated with GSTP1, Akt and ASK1 [40]. Moreover, GSTO1-1 has been identified as a crucial protein in the Toll-like receptor 4 (TLR4)-mediated pro-inflammatory pathway, such that its inhibition results in the relief of lipopolysaccharide (LPS)-stimulated inflammatory response. 

In conclusion, certain GSTs act as ligands or modulators of signaling kinases like JNK, ASK1, Akt, or receptors, RyRs and epidermal growth factor receptor (EGFR) [43,49,64,82,88]. Having in mind that a malignant phenotype is frequently followed by deregulated cell proliferation, through interaction with various signaling molecules, GSTs might also affect drug-resistance. Therefore, overexpressed GSTs act as caretakers, enabling cancer cells to develop resistance to anti-cancer drugs.

## 5. The Role of Glutathione Transferases in Chemoresistance: Potential Targets for Anti-Cancer Agents

Apart from pure contribution in the development of chemoresistance due to their conjugating activity, GSTs seem to interact with efflux transporters, in that way increasing anti-cancer drug efflux from the cell, another mechanism associated with the development of chemoresistance (reviewed in [1]). Indeed, synergistic interaction between GSTP1 and MRP-1 is shown to contribute to the development of resistance to ethacrynic acid, chlorambucil, vincristine and etoposide [1,92]. Similarly, GSTA1 contributes to chlorambucil chemoresistance [93], while through synergism of GSTM1 and MRP-1 cancer cells are protected from vincristine [94]. Therefore, catalytic, regulatory and synergistic roles of overexpressed GSTs might be considered as important contributing factors in at least three major chemoresistance mechanisms.

Since reversal of drug resistance may be, at least partially, achieved by molecules capable of inhibiting GSTs, a significant number of GST inhibitors have been synthesized, while certain natural inhibitors were also identified and investigated [1,80,95,96,97,98]. The majority of these molecules are either GST substrate or GSH analogues or mechanism-based inhibitors, therefore leading to enzyme inhibition in different ways. Taking advantage of the overexpression of specific GSTs in different cancers enables an efficient accumulation and/or activation of anti-cancer drug within the cancer cell. Indeed, for this reason GSTs are suitable as biomarkers for combination therapies with distinct GST inhibitors and for the development of novel anti-cancer drugs with targeted selectivity. Previously recognized as GST substrate, ethacrynic acid (EA) and its analogues were among the first investigated GST inhibitors [99,100]. They are shown to sensitize tumor cells to cytotoxic effects of alkylating agents; however, they also seem to exhibit significant side effects [6,101,102,103]. More promising results were obtained with ethacraplatin, a molecule of cisplatin coordinated with two EA ligands, and ethacraplatin-containing micelles (M-EA-Pt), shown to revert resistance to platinum based drugs in both GSTP1 and GSTT1 overexpression cells [104,105] Another compound enabling cells to overcome resistance to platinum based drugs, shown to inhibit GSTP1 enzyme activity, is auranofin, a gold-phosphine compound [106].

Glutathione analogs are also among GST targeting anti-cancer agents (Table 2). Indeed, different peptidase-stable GSH analogues were synthesized and tested as GSTA1, GSTM1 and GSTP1 competitive inhibitors [107,108,109]. The GSH-peptidomimetic that draws most attention is γ-glutamyl cysteinyl phenyl glycyl diethyl ester or TER199, also known as TLK199, a selective inhibitor of glutathione transferase P1-1. It acts on MAPK signaling pathway by disrupting JNK:GSTP1 protein-protein interaction, hence activating the kinase cascade [110]. Furthermore, TLK199 has been shown to potentiate the effect of various anti-cancer drugs since it also acts as an inhibitor of MDR-1, in that way more specifically affecting resistance to a range of anti-cancer drugs transported by this efflux transporter [29,111].

Another molecule able to disrupt GSTP1 protein-protein interaction with both JNK and TRAF2, is 6-(7-nitro-2,1,3-benzoxadiazol-4-ylthio)hexanol or NBDHEX [112]. This molecule is also considered a highly efficient GST suicide inhibitor, due to its ability to bind in the substrate binding site (H-site) of GSTP1 and form a complex with GSH (bound in G-site), in that way inhibiting GSTP1 enzyme activity. However, lack of specificity for GSTP1 due to higher affinity for GSTM2-2, limited its clinical application, leading to synthesis of NBDHEX analogues with improved selectivity for GSTP1 [45,46,113,114,115].

In the past few years, a diverse array of small molecules has been reported as GSTO1-1 inhibitors, many of them that had been developed without apparent knowledge of GSTO1-1 activity. Specifically, this class of GSTs possesses a functional cysteine residue in the catalytic center and for that reason renders more sensitive to generic thiol-alkylating agents [60,116]. Using novel screening techniques, Cravatt and associates identified a class of highly specific α-chloroacetamide inhibitors of GSTO1-1 that react irreversibly with cysteine in the active-site (e.g., ML175 and KT53) [117,118] Moreover, the observation that ML175, a specific GSTO1-1 inhibitor can inhibit LPS-stimulated inflammatory signaling, enables a novel approach in the development of anti-inflammatory drugs [119]. Another class of α-chloroacetamide compounds has been synthesized by Ramkumar and colleagues, among which C1-27 is recognized as the most potent GSTO1-1 inhibitor showing promising antitumor activity in both in vitro and in vivo models of colorectal cancer, without gross systemic toxicities [120]. Apoptotic cell selectivity, attributed to increased cell permeability during apoptosis, is observed for a small peptide sulfonate ester (NJP2) that irreversibly inhibits GSTO1-1 [121].

Interestingly, α-tocopherol (vitamin E), including several esterified tocopherols, such as (+)-α-tocopherol phosphate and (+)-α-tocopherol succinate are also potent inhibitors of both GSTP1-1 [126] and GSTO1-1 [127]. Among them, alpha tocopheryl succinate (α-TOS) is the most effective form of vitamin E analogues, affecting cancer cell death. Indeed, treatment with α-TOS shows promising results due to selective induction of apoptosis by mitochondrial destabilization [128]. It seems that the proton pump inhibitor omeprazole, used in treatment of gastroesophageal reflux and peptic ulcers, as well as, rifampicin, antibiotic that acts through inhibition of bacterial DNA-dependent RNA polymerase, also act as GSTO1-1 inhibitors [129,130,131]. What is more, in vivo experiments showed that oral pretreatment with omeprazole induces solid tumors sensitivity to chemotherapeutics [132]. It is important to note that these drugs are much less potent as inhibitors towards GSTO1-1, than the therapeutically relevant target. To date, most of the aforementioned compounds are still in need of substantial optimization before acquiring the qualities required for clinical trials.

Several natural products, such as aloe-emodin (anthraquinone from aloe vera leaves), benastatins (Aromatic polyketides from culture broths of *Streptomyces* species), certain flavonoids, plant polyphenols and alkaloids (e.g., piperlongumine from *Piper* species) have also been recognized as GST competitive inhibitors, some of them even being able to disrupt GSTP1:JNK complex [29,133,134,135]. Indeed, it seems that certain dietary agents are able to affect GSTP1 expression and epigenetic regulation. Namely, it has been shown that epigallocatechin-3-gallate, a polyphenol from green tea, can reverse epigenetically silenced GSTP1 gene in prostate cancer, while organosulfur compounds (e.g., garlic allyl sulfides) and sulforaphane rich cruciferous vegetables are able to increase expression and modulate activity of GSTP1 [136,137,138,139]. In this line, even compounds that act as histone deacetylase inhibitors are important for epigenetic regulation of GSTP1, since they are able to affect DNA hypermethylation in the promoter region of *GSTP1* gene and in that way induce transcription of *GSTP1* gene [140]. Regarding GSTO1-1, carnosic acid, a bio-active compound isolated from the herb Rosemary [80] and protoapigenone, a novel floavonoide isolated from *Thelypteris torresiana* [98] act as inhibitors.

The catalytic properties of GSTs might be exploited in a different manner when it comes to chemotherapeutics. Namely, there is a whole class of inactive cytotoxic agents named pro-drugs, which are converted into active drugs, or bio-activated, due to chemical modifications in enzyme catalyzed reactions [141]. The main role of these pro-drugs is to increase availability of anti-cancer drugs in target cells, while avoiding side effects in off-target ones. In other words, being highly selective in terms of izoenzymes that activate them, pro-drugs may accumulate in cancers cells with upregulated expression of that specific GST isoenzyme [1,97,142]. For that reason, pro-drugs with either GSH or GSH analogues and those whose activation demands GSH-conjugate intermediary compound are synthesized [143].

Among the first synthesized pro-drugs is a nitric oxide (NO) pro-drug [O^2^-{2,4-dinitro-5-[4-(*N*-methylamino)benzoyloxy]phenyl}1-(*N*,*N*-dimethylamino)diazen-1-ium-1,2-diolate) or PABA/NO, designed to release NO more readily when catabolyzed by GSTP1-1 in comparison to other GST isoenzymes [144,145]. Since NO present in high concentrations induces differentiation and apoptosis in cancer cells, a significant number of novel NO pro-drugs is being synthesized and investigated in vitro and in vivo [146,147]. One of NO pro-drugs shown to be efficient in solid tumors is another O^2^-(2,4-dinitrophenyl)diazeniumdiolates derivative named JS-K, which acts either by binding to GSTP1 with consequential release of high concentrations of NO or it binds to GST with previously bound GSH, decreasing its intracellular availability for detoxification reactions [148].

A pro-drug which has already reached phase III clinical trials is a modified glutathione analogue and nitrogen mustard pro-drug, TLK286 or canfosfamide. It is bio-activated by GSTP1-1 into alkylating metabolite capable of covalently binding DNA [143,149,150,151]. A great advantage of this promising GSTP-pro-drug is the fact that, either applied alone or in combination with conventional anti-cancer drugs, it shows no overlapping toxicity, no cross-drug resistance, and even has synergistic effect and last, but not least, it is well tolerated [1,142,152,153]. Another DNA binding drug that is also tested in clinical setting (phase II) is brostallicin [154,155,156]. Interestingly, this pro-drug is activated in reactions catalyzed by GSTP, but also GSTM, potentially enabling its application in tumors overexpressing either of the mentioned GST classes. 

A specific pro-drug has been identified even for cancer cells with upregulated GSTA1-1 expression. Namely, synthetic bombesin-sulphonamide derivatives are able to recognize bombesin receptor on cancer cell thus increasing drug uptake, which, once in the cell, undergoes GSTA1-1 catalyzed modification into GST competitive inhibitor [157].

Surprisingly, even metformin analogues are considered as GST pro-drugs. This drug, which is originally used in diabetes mellitus treatment, also exhibits certain anti-cancer effects [158] and is therefore considered a potential candidate in cancer treatment. Due to GST overrepression in cancer cells, few sulfonamide pro-drugs were synthesized, aiming GST catalyzed GSH-mediated amine formation form sulphonamide bonds [141,159,160].

The latest approach to treatment of solid tumors is development of pro-drugs that are derivatives of conventional anti-cancer drugs, such as doxorubicin (DOX). By incorporating sulfonamide moiety into existing anti-cancer drugs it becomes a pro-drug which, after being catalyzed by GSTs, releases the cytotoxic compound. In that way, a cytotoxic drug is released in high concentrations in cancer cells with upregulated GST expression, while cells with normal GST expression remain protected from afore mentioned cytotoxic effect [161]. Among these, 4-acetyl-2-nitro-benzenesulfonyl etoposide (ANS–etoposide) and 4-acetyl-2-nitro-benzenesulfonyl doxorubicin (ANS–DOX), function as pro-drugs for GSTA1. The more reactive 2,4-dinitrobenzenesulfonyl doxorubicin (DNS–DOX) showed preference for GSTP1 overexpressing cells. Additionally, these pro-drugs are even considered a shuttle system for DOX, and able to overcome resistance [161].

## 6. Conclusions

One of the major problems in conventional cancer therapy is the inability to selectively target cancer cells and to avoid side effects and chemoresistance to applied anti-cancer drug. Another important principle that needs to be respected is compliance with the novel approach in precision medicine, that the specific drug should be given in the specific dose to the specific patient. Glutathione transferases are responsible for both detoxification of numerous conventional chemotherapeutics, but also involved in regulation of cell proliferation and apoptosis. Due to their dual functionality and upregulated expression in various solid tumors they seem suitable for the development of novel drugs. Even more, different cancer cell types have a unique GST signature, enabling targeted selectivity for isoenzyme specific inhibitors and pro-drugs. The importance of GSTs substrate promiscuity was even contemplated based on the classical Greek aphorism “The fox knows many things, but the hedgehog knows one great thing” [162]. Namely, instead of being “hedgehogs” and able to catalyze only one reaction, GST are undoubtedly “foxes”, able to catalyze biotransformation of numerous substrates, including novel compounds with potential therapeutic efficacy. 

## Figures and Tables

**Table 1:** GST polymorphisms influence the drug resistance mechanisms of conventional anti-cancer drugs.

Anti-Cancer Drug	GST Class	GST Polymorphism Influencing Drug Response
***Detoxification by means of glutathione conjugation***		
**BCNU/Carmustine**	Alpha	Unknown [64,68]
	Mu	*GSTM1*, *GSTM3*–Unknown [29,68]
	Theta	*GSTT1*–Unknown [29,68]
**Busulfran**	Alpha, predominantly	GSTA1*B (-567G, -69T, -52A) [66,69]
**Brostallicin**	Alpha, Mu, Pi	Unknown [64,68]
**Carboplatin**	Pi, Alpha	Unknown [70]
**Chlorambucil**	Alpha	GSTA1*A (-567T, -69C, -52G) [1]
		*GSTA2*-2, point mutations in exon 5 and 7 [29,68]
	Pi	GSTP1*A (*Ile105/Ala114*) [61]
**Cisplatin, oxaliplatin**	Pi, Mu, Theta	Controversial [65]
**Cyclophosphamide**	Alpha	GSTA1*B (-567G, -69T, -52A) [29,68]
	Pi	GSTP1*B (*Val105/Ala114*) [29,68]
**Etoposide**	Pi	GSTP1*D (*Ile105/Val114*) [29,68]
**Melphalan**	Alpha	Unknown [63,64,68]
	Pi	GSTP1*D (*Ile105/Val114*) [29,68]
**Paclitaxel, docetaxel**	Pi	GSTP1*C (*Val105/Val114*) [29,68]
**Thiotepa**	Alpha	GSTA1*A (-567T, -69C, -52G) [29,68]
	Mu	Point mutation in exone 7 [29,68]
	Pi	GSTP1*A (Ile105/Ala114)
		*GSTP1* (*Ala114Val*) [67]
**Thiopurines**	Alpha, Mu	Unknown [64,68]
***Detoxification by means of redox regulation***		
**Doxorubicin**	Pi	*GSTP1*, point mutations in exons 5 and 6 [29]
**Other anthracyclines**	Alpha	*GSTA4–4*, Unknown [63]
Other anti-cancer drugs as GST substrates*		
	Yet to be determined	Unknown [68]

* bleomycin, dactinomycin, daunorubicin, fluorouracil, idarubicin, ifosfamid, mitomycin, mitoxantrone, vinblastine, vincristine, vinorelbine.

**Table 2:** GST inhibitors and pro-drugs with clinical perspective.

GST Inhibitors and Pro-Drugs	Mechanism	Clinical Perspective	Structure
**Ethacraplatin—containing micelles**	enhances the accumulation of active cisplatin in GSTP1 and GSTT1 overexpressing cancer cells by inhibiting the activity of GSTs and circumventing deactivation of cisplatin	with FDA-approved adjuvant, 1,2-distearoylsn-glycero-3-phosphoethanolamine-*N*-[methoxy(polyethylene glycol)-2000] (DSPE-PEG2000)	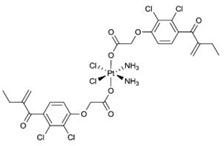 [122]
**TLK199**	selective inhibitor of GSTP1-1 acting on MAPK signaling pathway and inhibitor of MDR-1	completed or phase IIa clinical trial in non- small cell lung cancer and myelodysplastic syndrome	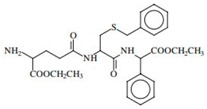 [72,73]
**Auranofin**	GSTP1 inhibitor which enables cells to overcome resistance to platinum-based drug	completed or recruiting phase II clinical trial in ovarian cancer, small and non-small cell lung carcinoma and lung adenocarcinoma	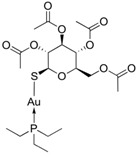 [123]
**TLK286**	bio-activation by GSTP1-1 into alkylating metabolite capable of covalently binding DNA	completed phase IIa and terminated phase III clinical trial in ovarian, breast and non-small cell lung cancer	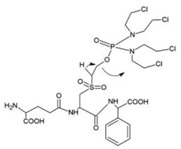 [124]
**Brostallicin**	activated in reactions catalyzed by GSTP and GSTM	completed phase II clinical trial in breast cancer	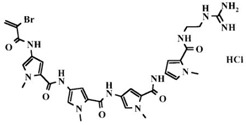 [125]

## Abbreviations

Akt	protein kinase B
ANS–etoposide	4-acetyl-2-nitro-benzenesulfonyl etoposide
ANS–DOX	4-acetyl-2-nitro-benzenesulfonyl doxorubicin
ASK1	apoptosis signal- regulating kinase
DHAR	dehydroascorbate reductase
DNS–DOX	2,4-dinitrobenzenesulfonyl doxorubicin
DOX	doxorubicin
EA	ethacrynic acid
EGFR	Epidermal growth factor receptor
GSH	glutathione
GST	glutathione transferases
GSTA	GST Alpha class
GSTM	GST Mu class
GSTO	GST Omega class
GSTP	GST Pi class
GSTS	GST Sigma class
GSTT	GST Theta class
GSTZ	GST Zeta class
JNK	c-Jun N-terminal kinases
JS-K	O^2^-(2,4-dinitrophenyl)diazeniumdiolates derivative
LPS	lipopolysaccharide
MAPK	mitogen-activated protein kinases
M-EA-Pt	ethacraplatin-containing micelles
MRP	multi-drug resistant protein
NBDHEX	6-(7-nitro-2,1,3-benzoxadiazol-4-ylthio) hexanol
NO	Nitric oxide
PABA/NO	[O^2^-{2,4-dinitro-5-[4-(N-methylamino)benzoyloxy]phenyl}1-(*N,N*-dimethylamino)diazen-1-ium- 1,2-diolate)
PG	prostaglandins
RyRs	ryanodine receptors
SNPs	single nucleotide polymorphisms
TER199	γ-glutamyl cysteinyl phenyl glycyl diethyl ester or TLK199
TLK286	canfosfamide
TLR4	Toll-like receptor 4
TRAF2	tumor necrosis factor receptor-associated factor 2
Trx	thioredoxin
